# Chlorine Dioxide Teat Disinfectant: A Clinical Study on Bactericidal Efficacy and Safety in Dairy Cows in Comparison with an Iodine Glycerin Disinfectant

**DOI:** 10.3390/ani16020312

**Published:** 2026-01-20

**Authors:** Jing Liu, Tingting Sun, Jiajia Wang, Huan Liu, Huanhuan Wang, Xiubo Li, Fei Xu

**Affiliations:** 1 National Feed Drug Reference Laboratories, Feed Research Institute, Chinese Academy of Agricultural Sciences, Beijing 100081, China; 2 Laboratory of Quality & Safety Risk Assessment for Products on Feed-origin Risk Factor, Ministry of Agriculture and Rural Affairs, Beijing 100081, China

**Keywords:** bovine mastitis, teat dip, chlorine dioxide, iodine glycerin, dairy safety

## Abstract

This study evaluated the clinical bactericidal efficacy and safety of a novel chlorine dioxide teat disinfectant compared to a traditional iodine glycerin disinfectant in dairy cows. The randomized controlled trial included long-term natural exposure (100 cows) and teat surface disinfection (40 cows) experiments. Key metrics assessed were somatic cell count (SCC), teat skin health (dryness, roughness, hyperkeratosis), and bacterial reduction rates against *Staphylococcus aureus*, *Escherichia coli*, and *Streptococcus* spp. Results demonstrated that the chlorine dioxide teat disinfectant achieved comparable to iodine glycerin disinfectant in controlling the rate of SCC exceeding the threhold (3.57% vs. 4.50% at day 10; *p* > 0.05), teat skin dryness, end roughness, and hyperkeratosis severity showed no significant differences over time or between iodine glycerin (control) and chlorine dioxide teat disinfectant groups (all *p* > 0.05). Notably, quantitative bacteriological assessment revealed significantly higher log_10_ reduction values for chlorine dioxide teat disinfectant (2.14) versus iodine glycerin controls (1.93; *p* < 0.05). Microbiological evaluation further demonstrated complete pathogen eradication (100.00%) by chlorine dioxide across all isolates (*S. aureus*, *E. coli*, *Streptococcus* spp.), whereas iodine glycerin achieved 99.84–100.00% bactericidal rates. The findings suggest that chlorine dioxide teat disinfectant is a sustainable and effective alternative to iodine glycerin disinfectant, offering robust antimicrobial activity, improved teat condition, and reduced residue concerns. Further multicenter studies are warranted to validate these outcomes under diverse herd management conditions.

## Simple Summary

Mastitis is a common disease in dairy cows caused by harmful bacteria, leading to eco-nomic losses in the dairy industry. This study compares the effectiveness and safety of two teat disinfectants: a new chlorine dioxide-based disinfectant and the traditional io-dine glycerin disinfectant. The study involved 100 cows in a long-term trial and 40 cows in a teat surface disinfection experiment. The results showed that both disinfectants were equally effective in reducing somatic cell counts and maintaining teat health. However, chlorine dioxide demonstrated a stronger bactericidal effect, completely eradicating harmful bacteria such as *Staphylococcus aureus*, *E. coli*, and *Streptococcus* spp. The findings suggest that chlorine dioxide is a promising alternative to iodine-based disin-fectants, offering similar or even better outcomes while being environmentally friendly and residue-free.

## 1. Introduction

Mastitis in dairy cows is primarily caused by pathogenic microorganisms such as bacteria, fungi, and viruses, which enter the mammary gland tissue through the teat duct or damaged skin, resulting in infection [1,2]. The global economic loss due to mastitis is estimated to be $22 billion [3]. Intramammary infection elevates somatic cell count (SCC) in milk, which subsequently alters its chemical composition due to enzymatic activity from white blood cells and bacteria; this leads to reduced lactose, mineral imbalances, and increased hydrolytic proteases, thus adversely affecting the flavour, shelf life, and processing properties of dairy products [4,5]. The common practice of using teat disinfectants before and after milking is an effective way to prevent mastitis because it reduces the amount of pathogens that enter the open teat canal, which lowers the rate of new infections and limits cross-contamination, especially in large-scale farms [6,7]. It also alleviates the dryness and cracking of teat skin caused by frequent milking and effectively reduces the microbial load on the surface of the teat skin, ensuring the safety of milk products [8].

Currently, common teat disinfectants used in the industry include iodine-based, chlorine-based, chlorhexidine-based, lactic acid-based, hydrogen peroxide-based, and quaternary ammonium compound-based teat disinfectants [9,10,11,12]. Iodine-based disinfectants such as povidone-iodine are most widely used due to their strong bactericidal effects, in dairy farms in China [13,14]. However, iodine is volatile, and the prolonged use of high-concentration iodine preparations may lead to excessive iodine content in the milk [15]. Chlorine dioxide, known as a fourth-generation disinfectant, possesses strong oxidizing properties and broad-spectrum antibacterial activity [16]. It has a fast bactericidal action, a wide bactericidal spectrum, and is environmentally friendly, without producing harmful by-products [17]. Research on chlorine dioxide teat disinfectants for dairy cows is limited in China. International studies on chlorine dioxide teat disinfectants show that, compared to iodine-based disinfectants, there is no significant difference in effectiveness [9,18]. Both can effectively reduce the bacterial load on the teat skin.

Two chlorine dioxide–based post-milking teat disinfectants, UDDER Gold (Ecolab) and Keno™mix (CID Lines), have not yet been launched in China. Publicly available technical documentation indicates that chlorine dioxide–based teat disinfectants have strong bactericidal activity and can support teat-skin condition; for example, UDDERgold products have been reported to reduce new intramammary infections in field testing, and Keno™mix has reported bactericidal performance according to EN 1656 as well as high emollient content for teat-skin conditioning. Given the limited variety of teat disinfectants currently available on Chinese dairy farms, our laboratory developed a chlorine dioxide–based teat disinfectant. The objective of this study was to compare the clinical bactericidal efficacy and safety of the newly developed chlorine dioxide teat disinfectant with those of a conventional iodine glycerin teat disinfectant.

## 2. Materials and Methods

### 2.1. The Selection of the Dairy Farm and the Selection of Experimental Dairy Cows

This experiment was conducted from March to April 2022 at the Nankou No. 2 farm of Beijing Capital Agribusiness Foods Group, located in Nankou Town, Changping District, Beijing. The farm is registered as a GCP-compliant experimental base. All dairy cow-related experiments were approved by the Institute of Feed Research, Chinese Academy of Agricultural Sciences. The farm houses about 800 lactating dairy cows, with bedding consisting of sand and sawdust. Milkings are performed three times (06:30, 14:30, 22:30) daily in a double 25-parallel milking parlor.

Prior to the experiment, milk samples from all lactating cows were collected from four quarters for somatic cell count (SCC) determination. A total of 100 lactating cows with SCC below 200,000 cells/mL in all four quarters were selected, and cows that had not received any antibiotic or anti-inflammatory treatment within the past 15 days were randomly divided into two groups for natural exposure trials. Additionally, 40 lactating cows with no prior antibiotic treatment and SCC below 200,000 cells/mL were selected, and after random division into two groups, they underwent a teat surface disinfection trial.

### 2.2. Sample Collection and SCC Measurement

Milk samples were taken from cows with reference to the Laboratory Handbook on Bovine Mastitis [19] (sample collection and handling 7–12, in the USA). Milk samples were collected during the second daily milking under strict aseptic conditions. Prior to collection, both the collector’s hands and udder teats underwent thorough disinfection using 70% ethanol, with subsequent removal of residual disinfectant via clean paper towels to ensure complete drying of skin surfaces. Approximately 25–30 mL of raw milk was aseptically collected into 50 mL sterile plastic centrifuge tubes at milking initiation, taking care to prevent teat contact with tube openings. Immediately following collection, samples were securely sealed with sterile caps and maintained at 4 °C throughout storage, with all microbiological analyses completed within 24 h of acquisition.

SCC was performed with a Fossmatic somatic cell detector (Foss Electric A/S, Hillerød, Denmark). The DNA was dye-stained in red, and the stained somatic cells were individually passed through the flow path system, emitting red light that was amplified by the photomultiplier, and finally counted by the light pulse capture. The instrument was calibrated using the Fossmatic adjustment before the sample was mounted.

### 2.3. Blinding and Allocation Concealment

The test and control disinfectants were prepared and relabeled as ‘Product A’ and ‘Product B’ by the project leader and one designated investigator, who were not involved in subsequent on-farm procedures. The allocation code was documented and sealed prior to trial initiation.

Milk sampling and pre-milking teat condition scoring were performed by a separate team of one primary investigator and three trained assistants, all of whom were blinded to treatment identity. Laboratory analyses, including somatic cell count and bacteriological examinations, were conducted by personnel blinded to group allocation until data lock and unblinding.

### 2.4. Natural Exposure Trial

The lactating cows selected for the natural exposure trial were 100 cows (400 quarters), which were randomly divided into two groups, with 50 cows (200 quarters) in each group. In the experimental group, after milking, the teats were immersed in chlorine dioxide teat disinfectant (produced by Hunan Xiaidi Biotechnology Co., Ltd., Changsha, Hunan province, China batch number YL211020) [20]. Before use, all the solution in Container A was poured into Container B and thoroughly mixed, and allowed to stand for 1 h (the effective concentration of chlorine dioxide can reach 135 mg/L). The disinfectant was applied externally for teat disinfection after milking in lactating cows. The control group was treated with iodine glycerin teat disinfectant (2% concentration, produced by Beijing Huaqinyuan Pharmaceutical Co. Ltd., Beijing, China, batch number 2109001). The application protocol for this disinfectant was as follows: the solution was diluted with 1 part of the disinfectant to 3 parts water for external use on teats post-milking (the effective iodine concentration is 0.5%, which is equivalent to 5 g/L).

After milking, both the control and experimental groups had their teats immersed in the respective disinfectants for 30 s. Milk samples were collected and SCC measured on days 0, 10, 20, and 30 as described in Section 2.2. The health status of the teats was observed on days 0, 10, 20, and 30, including scoring for skin dryness, roughness of the teat ends, and degree of keratinization [17,21].

### 2.5. Clinical Bactericidal Efficacy

The lactating cows selected for the natural bacterial teat disinfection trial were 40 cows, randomly divided into two groups, with 20 cows in each group. After milking, a sterile cotton swab was wetted with a neutralizing agent and used to wipe the left front teat from top to bottom, followed by wiping the right rear teat using the same swab with the same procedure. After completion, the cotton swab was placed in a 10 mL sterile centrifuge tube. The composite swabs from the left front and right rear quarters, collected prior to disinfection, were designated as PRE.

Subsequently, teat disinfectant was applied (the control group used iodine glycerin teat disinfectant, while the experimental group used chlorine dioxide teat disinfectant). The entire teat was immersed up to the base, and after 30 s, the same procedure was used to collect composite swabs from the right front and left rear quarters. These swabs were then placed in a 10 mL sterile centrifuge tube. The composite swabs collected after milking and disinfection were designated as POST.

The collected composite swabs were stored in a refrigerated box and transported back to the laboratory, where they were temporarily stored at 4 °C. Bacterial analysis was performed on the teat swabs to assess the total bacterial count, *Staphylococcus aureus*, *Escherichia coli*, and *Streptococcus* spp.

To process the samples, 2 mL of sterile saline was added to each centrifuge tube and vortexed for 1 min. Then, serial 10-fold dilutions were made up to the fourth dilution. A 0.1 mL aliquot from each dilution was plated on agar plates containing 5% fetal bovine serum TSA agar, eosin methylene blue agar, mannitol salt agar, and 5% sterile defibrinated sheep blood agar. The plates were incubated at 37 °C for 24 h, and colony counts were recorded for counts ranging from 25 to 250 colonies.

### 2.6. Statistical Analysis

For the udder health evaluation in the natural exposure trial, the proportion of quarters with elevated somatic cell count (SCC > 200,000 cells/mL) was calculated as:

N_SCC_/X_SCC_ × 100%,

where N_SCC_ represents the number of mammary quarters exceeding the SCC threshold and X_SCC_ denotes the total number of quarters examined at each time point (days 10, 20, and 30).

Clinical mastitis incidence was defined as the proportion of affected quarters (C_n_) relative to the total enrolled quarters (C_t_) at each time point (C_n_/C_t_ × 100%).

Teat condition was assessed using three parameters: teat skin dryness, teat-end surface roughness, and teat-end hyperkeratosis severity. For each parameter, the reduction rate (%) was calculated by comparing baseline scores at day 0 (T_0_) with scores at subsequent time points (T_x_; days 10, 20, or 30) using the formula:

[(T_0_ − T_x_)/T_0_] × 100%.

For the clinical bactericidal efficacy trial, bacterial reduction rate (%) and log_10_ reduction value were calculated for total bacterial counts, *Escherichia coli*, *Staphylococcus aureus*, and *Streptococcus* spp. The reduction rate was calculated as:

(1 − N_t_/N_0_) × 100%,

where N_0_ and N_t_ represent bacterial counts before and after disinfection, respectively. The log_10_ reduction value was calculated as:

log_10_(N_0_) − log_10_(N_t_).

Statistical analyses were applied according to the type of variable. Independent *t*-tests (unpaired) were used to compare proportions of SCC exceedance and clinical mastitis incidence between the chlorine dioxide teat disinfectant and iodine glycerin disinfectant groups at corresponding time points. One-way analysis of variance (ANOVA) was applied to teat skin dryness scores, teat-end surface roughness scores, teat-end hyperkeratosis scores, and bacterial counts to assess differences between treatment groups. When variance heterogeneity was detected using Levene’s test, Welch’s *t*-test was applied; otherwise, Student’s *t*-test (equal variances) or paired *t*-tests were used as appropriate for intergroup or intragroup comparisons. Bacterial reduction rates between disinfectant groups were compared using independent *t*-tests.

All results were expressed as percentages or mean ± standard deviation, and statistical significance was set at *p* < 0.05. All data processing, calculations, and statistical analyses were performed using Excel.

## 3. Results

### 3.1. Long-Term Natural Exposure Trial

A total of 100 lactating Holstein cows were selected for the long-term natural exposure trial and randomly divided into two groups, with 50 cows in each group (Table 1). On day 8 of the trial, one cow was excluded due to lameness, which prevented further participation. On days 15 and 22 of the trial, two cows were excluded on each day as they entered the dry period prematurely and could no longer continue the trial (Table 2).

**Figure 1:**
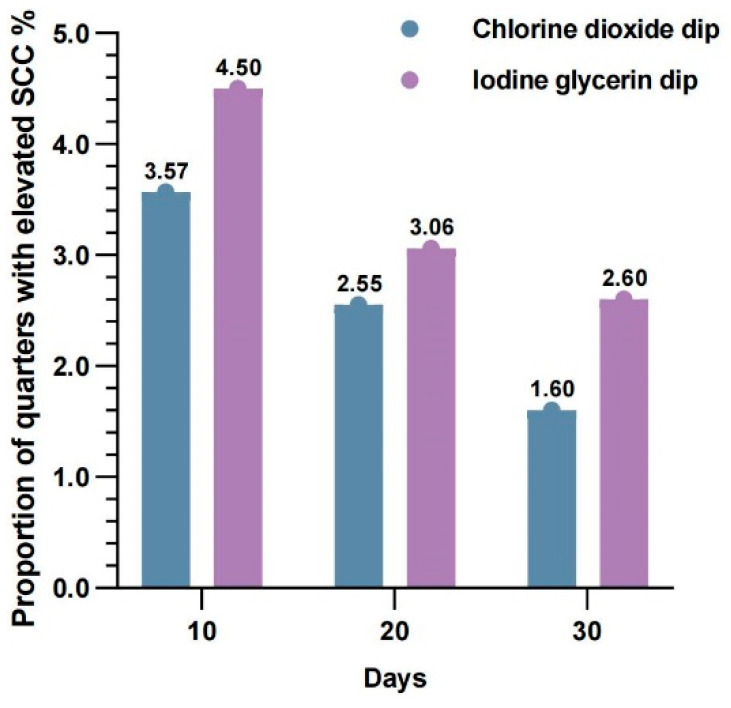
Proportion of quarters with elevated SCC in cows treated with chlorine dioxide teat disinfectant or iodine glycerin disinfectant at different exposure times. Elevated SCC was defined as SCC > 200,000 cells/mL.

The intergroup analysis of the proportion of quarters with elevated SCC above 200,000 cells/mL is illustrated in Figure 1. At days 10, 20, and 30 post-treatment, the SCC exceedance rates for the iodine glycerin disinfectant group were 4.500%, 3.061%, and 2.604%, respectively, while those for the chlorine dioxide teat disinfectant group were 3.571%, 2.551%, and 1.596%. No statistically significant differences were observed between the two groups at any timepoint (*p* = 0.499).

Throughout the trial period, both the iodine glycerin disinfectant group (control) and the chlorine dioxide teat disinfectant group maintained teat skin dryness scores ≤ 2. No statistically significant differences (*p* = 0.380) were observed between baseline (day 0) and days 10, 20, or 30 in either group (Table 3), indicating consistently low dryness levels. Furthermore, both teat end surface roughness (Table 4) and teat end hyperkeratosis severity (Table 5) exhibited non-significant intergroup variations (*p* = 0.362 and *p* = 0.287, respectively) throughout the study. Critically, intergroup analysis revealed no significant difference (Table 6) in teat skin dryness scores between the control and chlorine dioxide treatment groups.

These findings demonstrate that the chlorine dioxide teat disinfectant, when applied as a post-milking disinfectant, effectively maintained the proportion of quarters with elevated SCC below 3.571%, with no significant difference in efficacy compared to the iodine glycerin disinfectant (*p* > 0.05). The results suggest comparable performance between the two disinfectants in controlling subclinical mastitis risk under the tested conditions.

This analysis underscores the utility of chlorine dioxide-based formulations as a viable alternative to iodine-based products for teat hygiene management in dairy herds.

### 3.2. Clinical Bactericidal Efficacy

**Figure 2:**
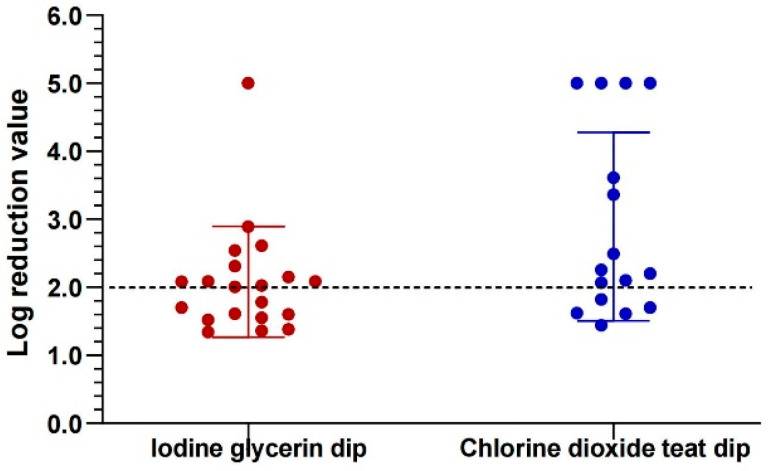
Comparative bactericidal log_10_ reductions for two teat disinfectants across 20 samples per group.

Quantitative bacteriological assessment of post-milking teat disinfection demonstrated (Figure 2) significantly higher log_10_ reduction values for chlorine dioxide (2.14) compared to iodine glycerin controls (1.93; *p* < 0.05 by F-test of baseline variances). Microbiological evaluation of teat disinfectants across 20 samples per group revealed that iodine glycerin disinfectant (Table S1) achieved mean bactericidal rates of 99.84% against *S. aureus* (5 isolates), 99.90% against *E. coli* (13 isolates), and 100.00% against *Streptococcus* spp. (14 isolates), whereas chlorine dioxide teat disinfectant (Table S2) demonstrated complete eradication (100.00%) against all pathogens, including *S. aureus* (6 isolates), *E. coli* (14 isolates), and *Streptococcus* spp. (16 isolates).

Pathogen-specific analyses were performed for major mastitis-associated bacteria, including *Staphylococcus aureus*, *Escherichia coli*, and *Streptococcus* spp. (Tables S1 and S2). In both treatment groups, near-complete elimination of these pathogens was observed following post-milking teat disinfection. Specifically, for *S. aureus*, bacterial counts were reduced to zero or near-zero levels in all positive samples in both the iodine glycerin and chlorine dioxide groups, with reduction rates ranging from 99.03% to 100%. Similar patterns were observed for *E. coli* and *Streptococcus* spp., where post-treatment counts were predominantly 0 cfu/mL.

## 4. Discussion

The study offers fresh proof of the effectiveness and safety of chlorine dioxide teat disinfectant for dairy cows, in comparison to the conventional iodine glycerin teat disinfectant. Compared to iodine glycerin, which had near-complete but marginally lower rates of eradication of all tested pathogens (*S. aureus*, *E. coli*, *and Streptococcus* spp.), chlorine dioxide produced significantly higher log_10_ reductions in total bacterial counts (2.14 vs. 1.93, *p* < 0.05) and complete eradication of all tested pathogens. Using an experimental challenge, Boddie [18] showed that teat disinfectants based on chlorine dioxide are as effective as those based on iodine in preventing new intramammary infections brought on by Streptococcus agalactiae and Staphylococcus aureus, and they do not compromise the integrity of the teat skin. Similar to this, Drechsler [22] found that products containing chlorine dioxide are superior to formulations containing 1% iodine in terms of lowering infections. Furthermore, Matti and Timms [23], using a split-udder design, confirmed that experimental chlorine-based teat disinfectants and commercial iodine disinfectants have no significant differences in maintaining teat skin and teat-end health, both supporting good skin condition. Collectively, these studies suggest that chlorine dioxide-based teat disinfectants can serve as an effective alternative to iodine-based disinfectants, offering comparable bactericidal efficacy and teat health protection [9,20].

No significant differences were observed between chlorine dioxide and iodine glycerin in controlling the rate of somatic cell count (SCC) exceeding standards or teat skin condition (dryness, roughness, hyperkeratosis). Both formulations demonstrated efficacy in reducing bacterial load on teat skin and maintaining udder health when applied correctly. Studies [8,22] indicate that post-milking disinfectants based on chlorine and iodine are equally effective in controlling SCC and promoting udder health, which aligns with the findings of the present study. Fitzpatrick [8] emphasized that mild disinfectant formulations are critical for maintaining teat skin integrity. This is consistent with the favorable tolerance profile observed in this study for both products, characterized by low rates of skin dryness or irritation. Furthermore, Timms [23], in a split-udder trial, reported no significant differences between chlorine-based and iodine-based post-milking disinfectants regarding teat skin and teat end health, with all teats maintaining good skin condition. Baumberger [24] also confirmed that chlorine dioxide and iodine-based pre-milking disinfectants exhibited comparable efficacy in reducing total bacterial counts on teat skin, although specific outcomes were influenced by farm management practices and disinfectant concentration. Collectively, these studies support the safety and effectiveness of both chlorine dioxide and iodine glycerin for post-milking teat care.

In addition to bactericidal efficacy and teat skin safety, the potential presence of chlorine-related residues in milk is an important consideration. Previous studies indicate that chlorine dioxide is highly reactive and chemically unstable, rapidly decomposing into chloride ions and other low-reactivity species, thereby limiting its persistence in food products. A systematic review by Haida et al. [17] reported that, under controlled application conditions, chlorine dioxide does not accumulate in food matrices and is unlikely to form stable or toxic residues at levels of regulatory concern. Nevertheless, direct residue determination remains essential for comprehensive food safety evaluation.

The similar field performance of chlorine dioxide (ClO_2_) and iodine observed in this study is biologically plausible. ClO_2_ acts as a strong oxidant, inactivating microorganisms through oxidation of cellular components, whereas iodine exerts antimicrobial effects via iodination and oxidative damage after penetration of free iodine [24,25]. Despite different chemical pathways, both cause rapid, non-specific microbial injury. Field efficacy may be lower than laboratory results because oxidizing agents can be quenched by organic matter and influenced by variable contact time on teat skin [25]. In addition, iodine formulations commonly include emollients such as glycerin, and NMC guidelines indicate that formulation and pH affect teat skin condition [19]. Collectively, these factors support ClO_2_ as a practical non-iodine alternative with comparable efficacy under farm conditions.

## 5. Conclusions

In this clinical trial, chlorine dioxide teat disinfectant demonstrated bactericidal efficacy and safety equivalent to, or exceeding, that of the traditional iodine glycerin disinfectant in dairy cows. Both products effectively controlled SCC and maintained excellent teat skin health; however, chlorine dioxide provided superior log reductions in pathogenic bacteria and complete pathogen eradication across all tested isolates. Importantly, chlorine dioxide’s favorable skin tolerability and residue-free profile offer significant advantages for sustainable mastitis prevention, addressing both animal welfare and milk quality concerns. These results support the use of chlorine dioxide as a practical, environmentally responsible alternative to iodine-based teat disinfectants in dairy production.

## Figures and Tables

**Table 1:** Descriptive statistical analysis of the experimental and control groups before the start of the trial.

	Iodine Glycerin Disinfectant (*n* = 50)			Chlorine Dioxide Teat Disinfectant (*n* = 50)		
	Maximum	Minimum	Average	Maximum	Minimum	Average
Parity	6.00	1.00	2.16	8	1	2.38
Lactation Period	399	79	173.85	486	51	159.10
SCC (×1000)	194	16	105.23	198	10	68.14
Milk yield (kg/milking)	63.20	23.90	40.69	68.90	18.10	44.19
Milk fat %	5.74	2.47	4.12	5.11	2.52	3.98
Protein %	3.52	2.72	3.19	4.19	2.68	3.20
Fat/Protein	1.99	0.74	1.30	1.65	0.79	1.24
Lactose %	5.47	4.87	5.18	5.47	4.57	5.22
Dry matter	14.81	11.32	13.20	14.48	11.76	13.11

Baseline data were obtained from the farm Dairy Herd Improvement (DHI) recording system prior to study initiation.

**Table 2:** Descriptive statistical analysis of the experimental and control groups after the start of the trial.

Cow Data	Control (*n* = 49)			Chlorine Dioxide (*n* = 48)		
	Maximum	Minimum	Average	Maximum	Minimum	Average
Parity	6.00	1.00	2.13	6	1	2.27
DIM	425	105	197.68	512	77	186.73
SCC (×1000)	1896	9	235.13	2181	10	187.71
Milk yield (kg/milking)	56.4	9.70	39.31	72.20	18.10	42.76
Milk fat %	5.92	2.47	3.92	5.52	2.02	3.97
Protein %	4.00	2.69	3.27	4.11	2.57	3.30
Fat/Protein	1.77	0.77	1.20	1.91	0.61	1.21
Lactose %	5.67	4.69	5.19	5.52	4.31	5.16
Dry matter	15.31	11.58	13.08	15.4	11.17	13.14

Baseline data were obtained from the farm Dairy Herd Improvement (DHI) recording system prior to study initiation.

**Table 3:** Scoring of teat skin dryness.

Days	Iodine Glycerin Disinfectant				Chlorine Dioxide Disinfectant			
	Total Score	Mean ± SD	Reduction Rate	*p* Value	Total Score	Mean ± SD	Reduction Rate	*p* Value
0	207	1.035 ± 0.184	-	-	208	1.040 ± 0.196	-	-
10	206	1.030 ± 0.171	0.483%	0.389	202	1.031 ± 0.172	0.903%	0.294
20	200	1.020 ± 0.141	1.410%	0.180	201	1.026 ± 0.158	1.393%	0.199
30	197	1.026 ± 0.159	0.866%	0.279	192	1.021 ± 0.144	1.800%	0.121

Teat skin dryness was scored using a standardized ordinal scale, with lower scores indicating better teat skin condition. All scoring assessments were performed by trained personnel following the same criteria throughout the study.

**Table 4:** Scoring of teat end surface roughness.

Days	Control				Chlorine Dioxide			
	Total Score	Mean ± SD	Average Score Reduction Rate	*t* Test *p* Value	Total Score	Mean ± SD	Average Score Reduction Rate	*t* Test *p* Value
0	207	1.035 ± 0.184	-	-	207	1.035 ± 0.184	-	-
10	205	1.025 ± 0.156	0.966%	0.279	201	1.026 ± 0.158	0.917%	0.279
20	200	1.020 ± 0.141	1.410%	0.180	200	1.020 ± 0.141	1.410%	0.180
30	196	1.021 ± 0.143	1.369%	0.180	191	1.016 ± 0.125	2.661%	0.100

Teat-end surface roughness was assessed using a standardized visual scoring scale, with lower scores indicating smoother teat-end surfaces.

**Table 5:** Grading of teat end hyperkeratosis severity.

Days	Control				Chlorine Dioxide			
	Total Score	Mean ± SD	Reduction Rate	*p* Value	Total Score	Mean ± SD	Average Score Reduction Rate	*p* Value
0	204	1.020 ± 0.140	-	-	204	1.020 ± 0.140	-	-
10	203	1.015 ± 0.122	0.490%	0.352	198	1.010 ± 0.100	0.960%	0.206
20	198	1.010 ± 0.100	0.960%	0.206	198	1.010 ± 0.100	0.960%	0.206
30	194	1.010 ± 0.102	0.940%	0.206	189	1.005 ± 0.073	1.439%	0.089

Teat-end hyperkeratosis severity was evaluated using an ordinal scoring system, with lower scores indicating milder keratinization.

**Table 6:** Intergroup comparative analysis of evaluation metrics for chlorine dioxide teat disinfectant and iodine glycerin disinfectant across timepoints.

Assessment Criteria	Days			
	0	10	20	30
Scoring of teat skin desiccation severity	0.396	0.486	0.368	0.380
Scoring of teat end surface roughness	0.500	0.487	0.500	0.362
Grading of teat end hyperkeratosis severity	0.500	0.335	0.500	0.287

## Data Availability

Data are contained within the article.

## Supplementary Materials

The following supporting information can be downloaded at: https://www.mdpi.com/article/10.3390/ani16020312/s1, Table S1: Bacterial reduction rate before and after application of iodine glycerin teat dip; Table S2: Bacterial reduction rate before and after application of chlorine dioxide teat dip.
