# Supplementary material for: Chlorine Dioxide Teat Disinfectant: A Clinical Study on Bactericidal Efficacy and Safety in Dairy Cows in Comparison with an Iodine Glycerin Disinfectant
Source: Animals (Basel). 2026 Jan 20;16(2):312. doi: 10.3390/ani16020312 (PMC12837152; doi:10.3390/ani16020312)
Supplement: Supplementary file 1 [file animals-16-00312-s001.zip › animals-4064839-supplementary.pdf]

Table S1 Bacterial reduction rate before and after application of iodine glycerin teat dip.

NO.	Cow ID	*Staphylococcus aureus*（cfu/mL）			*Escherichia coli*（cfu/mL）			*Streptococcus spp.*（cfu/mL）		
		PRE	POST	Bacterial reduction rate	PRE	POST	Bacterial reduction rate	PRE	POST	Bacterial reduction rate
1	170397	125	0	100.00%	-	-	-	750	0	100.00%
2	170183	-	-	-	330	0	100.00%	65	0	100.00%
3	150438	105	0	100.00%	-	-	-	72	0	100.00%
4	180229	-	-	-	294	1	99.66%	-	-	-
5	170192	75	0	100.00%	216	0	100.00%	105	0	100.00%
6	170210	-	-	-	72	0	100.00%	50	0	100.00%
7	180227	-	-	-	45	0	100.00%	-	-	-
8	150279	-	-	-	-	-	-	260	0	100.00%
9	170239	64	0	100.00%	104	0	100.00%	320	0	100.00%
10	180290	-	-	-	-	-	-	25	0	100.00%
11	170137	-	-	-	25	0	100.00%	-	-	-
12	160490	-	-	-	-	-	-	126	0	100.00%
13	180314	-	-	-	75	0	100.00%	270	0	100.00%
14	170131	-	-	-	105	0	100.00%	136	0	100.00%
15	130087	-	-	-	-	-	-	-	-	-
16	160093	245	2	99.18%	188	0	100.00%	640	0	100.00%
17	170204	-	-	-	515	5	99.03%	-	-	-
18	160390	-	-	-	-	-	-	285	0	100.00%
19	130543	-	-	-	25	0	100.00%	-	-	-
20	170118	-	-	-	105	0	100.00%	125	0	100.00%

Table S2 Bacterial reduction rate before and after application of chlorine dioxide teat dip.

NO.	Cow ID	*Staphylococcus aureus*（cfu/mL）			*Escherichia coli*（cfu/mL）			*Streptococcus spp.*（cfu/mL）		
		PRE	POST	Bacterial reduction rate	PRE	POST	Bacterial reduction rate	PRE	POST	Bacterial reduction rate
1	180098	-	-	-	-	-	-	-	-	-
2	180392	175	0	100.00%	60	0	100.00%	130	0	100.00%
3	151522	-	-	-	-	-	-	35	0	100.00%
4	140513	45	0	100.00%	-	-	-	120	0	100.00%
5	180078	-	-	-	110	0	100.00%	750	0	100.00%
6	180018	65	0	100.00%	960	0	100.00%	100	0	100.00%
7	170421	-	-	-	95	0	100.00%	30	0	100.00%
8	190058	-	-	-	15	0	100.00%	-	-	-
9	190019	-	-	-	25	0	100.00%	800	0	100.00%
10	190073	-	-	-	65	0	100.00%	-	0	-
11	170047	-	-	-	25	0	100.00%	10	0	100.00%
12	180423	-	-	-	-	-	-	300	0	100.00%
13	180348	1025	0	100.00%	15	0	100.00%	650	0	100.00%
14	151475	5	0	100.00%	70	0	100.00%	-	-	-
15	170043	-	-	-	-	-	-	250	0	100.00%
16	190066	755	0	100.00%	15	0	100.00%	250	0	100.00%
17	190084	-	-	-	10	0	100.00%	1250	0	100.00%
18	190093	-	-	-	-	-	-	500	0	100.00%
19	190052	-	-	-	5	0	100.00%	300	0	100.00%
20	190082	-	-	-	635	0	100.00%	210	0	100.00%
